# Preoperative anxiety and its associated factors among women undergoing elective caesarean delivery: a cross-sectional study

**DOI:** 10.1186/s12884-022-04979-3

**Published:** 2022-08-17

**Authors:** Yewlsew Fentie, Tikuneh Yetneberk, Moges Gelaw

**Affiliations:** Departement of Anesthesia, College of Medicine and Health Sciences, Debre Tabor University, P.O.Box: 272, Debre Tabor, Ethiopia

**Keywords:** Anxiety, Cesarean delivery, Elective, Factors, Preoperative

## Abstract

**Background:**

Anxiety is a behavioral expression of tension and unpleasant emotion that arises from multifactorial dimensions that might increase the mortality of patients during anesthesia and surgery. This study aimed to verify the prevalence and associated factors of preoperative anxiety among women undergoing elective cesarean delivery.

**Method:**

A cross-sectional study design was conducted on a total of 392 patients who underwent elective cesarean delivery in Debre Tabor Comprehensive Specialized Hospital, in North Central Ethiopia from October 15, 2020, to September 15, 2021. Data was collected using a validated Amsterdam questionnaire, after translating to the local language (Amharic). Descriptive statistics were expressed in percentages and presented in tables. Bivariable and multivariable logistic analyses were done to identify factors associated with preoperative anxiety. The statistical significance level was set at *P* < 0.05 with 95% CI.

**Results:**

The overall prevalence of preoperative anxiety in women undergoing elective cesarean delivery was 67.9 [95% CI = (63.0–72.7)]. Participants who came from rural areas [AOR = 2.65; 95%CI: 1.27–5.53], farmers [AOR = 2.35; 95%CI: 1.02–5.40], participants with no previous surgical and anesthesia history [AOR = 2.91; 95%CI: 1.69–5.01], and primiparous women [AOR = 1.69; 95%CI: 1.01–2.83] were more significantly associated with preoperative anxiety.

**Conclusion:**

The prevalence of preoperative anxiety among elective cesarean deliveries was found to be high. So, preoperative maternal counseling and anxiety reduction services should therefore be given top priority, particularly for those women who came from rural areas, are farmers, have no prior surgical or anesthetic experience, and are primiparous.

**Supplementary Information:**

The online version contains supplementary material available at 10.1186/s12884-022-04979-3.

## Introduction

Anxiety is a behavioral expression of tension and unpleasant emotion that arises from multifactorial dimensions that can be divided into state and trait anxiety [1, 2]. State anxiety is acute situational anxiety or temporary situational anxiety triggered by a driving event that doesn’t persist after the factor is no more continuous but, trait anxiety is a personality manifestation that persists in lifelong patterns [1].

The incidence of preoperative anxiety during cesarean delivery is high compared to other surgical procedures especially in developing countries [3, 4]. The reports of different studies showed that 73.3% to 86% of women undergoing cesarean delivery experience preoperative anxiety [5, 6]. Despite the high burden of cesarean deliveries in Ethiopia, the preoperative anxiety is almost unforeseen. This leads to avoidable but unalleviated suffering for the women [7].

Preoperative anxiety is one of the causes of increased mortality of patients during anesthesia and surgery [3]. It has a great impact on the hemodynamics of patients that leads to an unnecessary increase in preoperative heart rate and blood pressure [8] causing acute myocardial infarction, heart failure, and pulmonary edema which lead to and high rate of cardiac mortality. preoperative anxiety can also increase the incidence of hypotension after spinal anesthesia [9] causing a need to give extra fluid and vasopressors [10].

Preoperative anxiety can lead women to refuse cesarean delivery which causes fatal events in the fetus and the mother other than anxiety-related complications [5, 8]. It also influences neonatal outcomes causing a decreased Appearance, Pulse, Grimace, Activity, and Respiration (APGAR) score [4]. Preoperative anxiety is a major cause of readmission [3], hospital stay, infection, increased analgesic requirement, and cost of hospitals that reduces overall maternal satisfaction with perioperative hospital services [3, 11, 12, 13].

It is critical for medical professionals and hospitals in underdeveloped countries to establish safe obstetric care services to prevent maternal fatalities caused by risks associated with pregnancy and childbirth [14]. Maternal death is most common in Africa, and the World Health Organization's current advice is to reduce death by providing safe obstetric surgery and anesthesia [15].

Given limited studies of preoperative maternal anxiety in Ethiopia and clinical observations, this study aimed to verify the prevalence and associated factors of preoperative anxiety among women undergoing elective cesarean delivery in Debre Tabor Comprehensive Specialized Hospital (DTCSH). This helps to identify the current gap and enhance the cesarean delivery care quality.

## Methods and materials

### Study design, area, and period

The cross-sectional study was conducted in DTCSH which is a public hospital established in 1934 and located in the south Gondar zone of Amara regional state at 667 km Northwest of Addis Ababa, the capital city of Ethiopia. It is 97 km to the southwest of Bahir Dar, the capital city of Amara regional state. Based on the information obtained from the south Gondar health department evidence of 2021, Debre Tabor Town has a total population of 87,627(45,670 females and 41,957 males) and about 53,416 of the population (27,402 females vs. 26,014 males) were in the reproductive age group. It has a latitude and longitude of 11051N3801’E11.8500 N 38.0170E with an elevation of 2,706 m (8878ft) above sea level. The prevalence of overall cesarean delivery in DTCSH is 39.1% [16] and based on data from the chart review, about 438 elective cesarean deliveries were performed per year. Seven operation rooms give service and of those, three tables for general surgery, two tables for orthopedic surgeries, and two tables for gynecology and obstetrics surgeries. The study was conducted on women who gave birth with elective cesarean delivery at DTCSH from October 15, 2020, to September 15, 2021.

### Inclusion criteria and exclusion criteria

All women who gave birth with elective cesarean delivery in DTCSH were included whereas; Patient refusal, Patients with psychological disorders, Language barrier, cesarean delivery with mothers and fetus are under life-threatening problems, psychological trauma, mothers taking any anxiolytic, ASA III and ASA IV were excluded.

### Sample size and sampling technique

The sample size was determined by using single proportion population formula taking 50% (P) of the proportion using a 95% confidence interval and 5% margin of error (d). The sample size was determined using the following formula.

$$n={\left({\mathrm{Z}}_{a/2}\right)}^{2}\mathrm{P}\left(1-\mathrm{P}\right)/{\mathrm{d}}^{2}$$

$$n= [(1.96)2 0.5(1-0.5)2] / (0.05)2$$

*n* = 385 then by adding 5% non-respondent rate.

*n* = 385 + 20 = 405 this was the final sample size.

The non-probable convenient sampling technique was applied until the intended sample size was achieved on elective cesarean deliveries.

### Data collection instrument and procedures

Data were collected using a structured questionnaire in the evening a day before cesarean delivery for all consecutive elective cesarean deliveries. A short version of the State-Trait Anxiety Inventory Scale (STAIS) was used to assess the outcome variable, which was adapted from a previously validated questionnaire [25]. Although the original STAIS is valid and reliable, with a Cronbach's alpha of 0.896, it has 40 questions, with 20 items for each subscale (state and trait), making data collection more difficult [17, 18]. The short form of STAIS is a widely used validated tool and more effective than the original version with Cronbach’s alpha = 0.896–0.950, and it is made up of six items taken from the original version (Table 1). In addition, the tool is appropriate for anyone aged 15 and above [18–20]. The questionnaire was primarily prepared in the English language then three language experts translated the English version of the tool into the Amharic local language, and the other three language experts translated it back to the English language. For data collection, three anesthetists were assigned.

**Table 1:** Short version of STAIS-tool

Items	Almost never	Sometimes	often	Almost always
Negative Items				
I feel tense	1	2	3	4
I feel upset	1	2	3	4
I feel worried	1	2	3	4
**Positive Items**				
I feel calm	4	3	2	1
I feel relaxed	4	3	2	1
I feel content	4	3	2	1

### Data quality assurance

To assure the quality of the data, the data collecting tool (the questionnaire) was pre-tested on 5% of study participants who were not involved in the main study at Felege Hiwot Comprehensive Specialized Hospital in Bahir Dar. Data collectors were given training for two days, and data was collected and appropriately filled in the prepared format. Throughout the data collection period, supervision was performed to ensure that the collected data was accurate, clear, and consistent.

### Data entry and analysis

Data were cleaned, coded, and entered into Epi data version 4.2 and exported to SPSS version 23 for analysis of the data. Descriptive statistics were carried out and the result was presented using text and tables. Both bi-variable and multivariable logistic regression analyses were used to identify factors associated with the anxiety level of women undergoing elective cesarean delivery. Variables with a *p*-value of less than < 0.2 in the Bivariable logistic analysis were fitted into multivariable logistic regression analysis. Both Crude Odds Ratio (COR) in bivariable logistic regression and Adjusted Odds Ratio (AOR) in multivariable logistic regression with the corresponding 95% Confidence interval were calculated show association and strength of association respectively. In multivariable logistic regression analysis, variables with a *p*-value of < 0.05 were considered statistically significant. To check for the goodness of fit, the Hosmer–Lemeshow goodness of fit test was used, with a *p*-value of 0.784.

### Operational definitions

#### Anxious

Respondents with STAIS scores of greater than or equal to 44 were considered anxious preoperatively [18–20].

#### Not anxious

Respondents with STAIS score of less than 44 were considered not anxious preoperatively [18–20].

## Result

In this study, 405 women were involved while 392 participants were enrolled in the study with a response rate of 96.8%. Thirteen women were excluded from the analysis due to incomplete data. About half of the study participants (50.3%) were in the age range of 18–29 years, 81.4% were married, and majorities (58.4%) were from rural areas (Table 2).

**Table 2:** Socio-demographic characteristics of study participants undergoing elective cesarean deliveries (*n* = 392)

Variables	Frequency (n)	Percentage (%)
**Age(years)**		
18–29	197	50.3
30–39	149	38.0
40 and above	46	11.7
**Residency**		
Urban	163	41.6
Rural	229	58.4
**Religion**		
Orthodox Christian	209	53.3
Muslims	103	26.3
Protestant	80	20.4
**Marital status**		
Married	319	81.4
Not married	73	18.6
**Educational level**		
Illiterate	191	48.7
Elementary	98	25.0
Secondary	64	16.3
College degree and above	39	10.0
**Profession**		
Housewife	47	12.0
Farmer	117	29.9
Student	75	19.1
Private employ	86	21.9
Government employ	67	17.1
**Annual income (ETB)**		
Less than 3781	275	70.2
3781 and above	117	29.8
**Surgical and anesthetic history**		
Yes	162	41.3
No	230	58.7
**Parity**		
Prime	165	42.1
Multi	227	57.9
**Gestational age**		
Preterm	64	16.3
Term	202	51.5
Post-term	126	32.2
**Indication**		
Previous cesarean delivery	97	24.7
Malposition	85	21.7
Major degree placenta previa	22	5.6
Hypertensive disorders of pregnancy	66	16.8
Fetal macrosomia	49	12.5
Oligohydramnios	30	7.7
Others	43	11.0

### The prevalence and possible causes of preoperative anxiety

The overall prevalence of preoperative anxiety in women undergoing elective cesarean delivery was 67.9 [95% CI = (63.0–72.7)]. From descriptive analysis fear of death (85.2%) was the major possible cause of preoperative anxiety. While, Family issues, Dependency, Cosmetic issues, Awareness during anesthesia & Cesarean delivery, Fear of experience with gynecologists/integrated emergency surgery and obstetrics (IESOs), Fear of experience with anesthetists, Information from previous negative hospital experiences, Lack of recognition of staff, and Bad obstetric history of previous Delivery were possible causes of preoperative anxiety less than 50% (Table 3).

**Table 3:** Possible causes of preoperative anxiety among study participants undergoing elective cesarean deliveries (*n* = 392)

Possible causes of preoperative anxiety	Yes n (%)	No n (%)
Fear of Death	334(85.2)	58(14.8)
Fear of unexplained origin	263(67.1)	129(32.9)
Financial loss	113(28.8)	279(71.2)
Family issues	224(57.1)	168(42.9)
Postoperative pain	256(65.3)	136(34.7)
Dependency	157(40.1)	235(59.9)
Disability	247(63.0)	145(37.0)
Complications	309(78.8)	83(21.2)
Cosmetic issues	134(34.2)	258(65.8)
Medical mistakes	306(78.1)	86(21.9)
Unable to recover from anesthesia	201(51.3)	191(48.7)
Awareness during anesthesia & Cesarean delivery	124(31.6)	268(68.4)
Fear of experience with a gynecologist/ integrated emergency surgery and obstetrics professionals	144(36.7)	248(63.3)
Fear of experience anesthetist	118(30.1)	274(69.9)
Information from previous negative hospital experiences	172(43.9)	220(56.1)
Lack of recognition of staff	195(49.7)	197(50.3)
Bad obstetric history of previous Delivery	37(9.4)	355(90.6)

### Factors associated with a preoperative anxiety level of study participants undergoing elective Cesarean deliveries

In this study, the bivariable logistic regression showed that residency, educational level, profession, surgical & anesthetic history, parity, fear of death, postoperative pain, fear of complications, fear of unrecovered from anesthesia, fear of disability, and fear of medical mistakes were factors with a *p*-value of less than 0.2 and were fitted with a multivariable logistic regression model. However, educational level and fear of disability were not associated in multivariable logistic regression with a *p*-value greater than 0.05.

In multivariable logistic analyses study participants who came from rural areas [AOR = 2.65; 95%CI: 1.27–5.53], farmers [AOR = 2.35; 95%CI: 1.02–5.40], participants with no previous surgical and anesthesia history [AOR = 2.91; 95%CI: 1.69–5.01], and primiparous women [AOR = 1.69; 95%CI: 1.01–2.83] were more significantly associated with preoperative anxiety. Also, study participants with Fear of death [AOR = 2.29; 95%CI: 1.13–4.67], fear of postoperative pain [AOR = 1.82; 95%CI: 1.10–3.02], fear of complications [AOR = 3.75; 95%CI: 1.83–7.67], fear of medical mistakes [AOR = 2.53; 95%CI:1.33–4.80] and fear of unable to recover from anesthesia [AOR = 3.58; 95%CI: 2.16,5.93] were more likely anxious than their counterparts (Table 4).

**Table 4:** Factors associated with preoperative anxiety of study participants undergoing elective CDs (*n* = 392)

Variables	Preoperative anxiety level		Crude odds ratio	Adjusted odds ratio	*p*-value
	Anxious	Not Anxious	(95% CI)	(95% CI)	
**Residency**					
Urban	119(73.0%)	44(27.0%)	1	1	
Rural	147(64.2%)	82(35.8%)	0.66(0.43,1.03)	2.65(1.27,5.53)	0.010*
**Profession**					
Housewife	37(78.7%)	10(21.3%)	1.12(0.45,2.83)	0.56(0.19,1.64)	0.292
Farmer	68(58.1%)	49(41.9%)	2.99(1.47,6.08)	2.35(1.02,5.40)	0.044*
Student	51(68.0%)	24(32.0%)	1.96(0.90,4.25)	1.61(0.66,3.93)	0.299
Private employ	56(65.1%)	30(34.9%)	2.25(1.05,4.71)	1.48(0.65,3.36)	0.356
Government employ	54(80.6%)	13(19.4%)	1	1	
**Surgical & anesthetic history**					
Yes	130(80.2%)	32(19.8%)	1	1	
No	136(59.1%)	94(40.9%)	2.81(1.76,4.48)	2.91(1.69,5.01)	0.000*
**Parity**					
Prime	96(58.2%)	69(41.8%)	2.14(1.39,3.29)	1.69(1.01,2.83)	0.047*
Multi	170(74.9%)	57(25.1%)	1	1	
**Fear of Death**					
Yes	220(65.9%)	114(34.1%)	1.99(1.01,3.89)	2.29(1.13,4.67)	0.022*
No	46(79.3%)	12(20.7%)	1	1	
**Postoperative pain**					
Yes	168(65.6%)	88(34.4%)	1.35(0.86,2.13)	1.82(1.10,3.02)	0.020*
No	98(72.1%)	38(27.9%)	1	1	
**Complications**					
Yes	196(63.4%)	113(36.6%)	3.10(1.64,5.86)	3.75(1.83,7.67)	0.000*
No	70(84.3%)	13(15.7%)	1	1	
**Medical mistakes**					
Yes	200(65.4%)	106(34.6%)	1.75(1.01,3.04)	2.53(1.33,4.80)	0.005*
No	66(76.7%)	20(23.3%)	1	1	
**Unable to recover from anesthesia**					
Yes	121(60.2%)	80(39.8%)	2.08(1.35,3.22	3.58(2.16,5.93)	0.000*
No	145(75.9%)	46(24.1%)	1		

^*^ *p*-value < 0.05 1 = reference

## Discussion

Preoperative anxiety is a predictable and manageable problem in women that undergoes elective cesarean delivery [21]. Women undergoing elective cesarean delivery may result in a variety of perioperative complications due to inappropriate preoperative consultation and poor treatment strategies for anxiety [22, 23].

In this study, 67.9% of women undergoing elective cesarean delivery were anxious in the perioperative period. This result was similar to a study done in Pakistani, in which preoperative anxiety was 72.7% [5]. On contrary, this result was higher compared to a study done in India (55%) [6] and Spain (9.2%) [24]. The possible causes of the high prevalence of preoperative anxiety in our setup might be due to lack of specific anxiety prevention and management protocol in the obstetrics ward, and limited awareness among professionals compared with the above study areas.

The multivariable logistic analyses showed that women who came from rural areas, no previous surgical and anesthesia history, primiparous, with Fear of death, with fear of postoperative pain, and with fear of complications were more anxious preoperatively than their counterparts.

In our study women who came from rural areas were more anxious preoperatively than those who came from urban. This might be due to the awareness level about cesarean delivery and related risks and benefits of women from rural areas might be less compared with urban.

In this study women who had no prior surgical or anesthetic history were more anxious than women with previous surgical and anesthetic history. The findings were comparable to those of studies conducted in India [6] and Colombia [24]. The possible explanation for this might be that previous experience might reduce anxiety from unknown origin and reduces misconceptions.

Parity was one of the determining factors for preoperative anxiety and primiparous women were more anxious than multiparous women. This finding was comparable to a study done in Pakistan study [5]. The possible reason for this might be multiparous women can be aware of the risks and consequences of childbirth which will reduce the anxiety level of unknown origin or complications in primiparous. However, a study conducted in Turkey showed that parity did not a significant factor for preoperative anxiety [25].

Preoperative anxiety was substantially correlated with cesarean delivery-related outcomes such as fear of death, fear of postoperative pain, fear of complications, fear of medical mistakes, and fear of being unable to recover from anesthesia. Studies conducted in Australia [26] and the United Kingdom [27] support this finding. The possible explanation for this might be that the fear of negative outcomes can produce an increased level of anxiety.

### Limitations of the study

The study was conducted during the COVID-19 outbreak and war in the country which could have affected the level of preoperative maternal anxiety done by elective cesarean delivery.

## Conclusion

The prevalence of preoperative anxiety among elective cesarean deliveries was found to be high. So, preoperative maternal counseling and anxiety reduction services should therefore be given top priority, particularly for those women who came from rural areas, are farmers, have no prior surgical or anesthetic experience, and are primiparous.

## Supplementary Information

**Additional file 1.**

## Data Availability

The datasets used and/or analyzed during the current study are available from the corresponding author upon reasonable request.

## Abbreviations

APGAR: Appearance, Pulse, Grimace, Activity, Respiration
ASA: American Society of Anesthesiology
CD: Cesarean Delivery
DTCSH: Debre Tabor Comprehensive Specialized Hospital
ETB: Ethiopian Birr
STAIS: State-Trait Anxiety Involuntary scale
WHO: World Health Organization
