# Supplementary material for: Preoperative anxiety and its associated factors among women undergoing elective caesarean delivery: a cross-sectional study
Source: BMC Pregnancy Childbirth. 2022 Aug 17;22:648. doi: 10.1186/s12884-022-04979-3 (PMC9382617; doi:10.1186/s12884-022-04979-3)
Supplement: Supplementary file 1 — Additional file 1. [file 12884_2022_4979_MOESM1_ESM.docx]

**Questionnaire for Preoperative Anxiety and Its Associated Factors among Women Undergoing Elective Caesarean Delivery: A Cross-sectional study**

1. **Demographic and clinical characteristics of study participants (circle one)**

| **Code** | **Questioner** | **characteristics** |
| --- | --- | --- |
| 101 | Age(years) | 1. 18-29 2. 30-39 3. 40 and above |
| 102 | Residency | 1. Urban 2. Rural |
| 103 | Religion | 1. Orthodox Christian 2. Muslims 3. Protestant |
| 104 | Marital status | 1. Married 2. Not married |
| 105 | Educational level | 1. Illiterate 2. Elementary 3. Secondary 4. College degree and above |
| 106 | Profession | 1. Housewife 2. Farmer 3. Student 4. Private employ 5. Government employ |
| 107 | Annual income (ETB) | 1. Less than 3781 2. 3781 and above |
| 108 | Surgical and anesthetic history | 1. Yes 2. No |
| 109 | Parity | 1. Prime 2. Multi |
| 110 | Gestational age | 1. Preterm 2. Term 3. Post-term |
| 111 | Indication | 1. Previous cesarean delivery 2. Malposition 3. Major degree placenta previa 4. Hypertensive disorders of pregnancy 5. Fetal macrosomia 6. Oligohydramnios 7. Others |

1. **Possible causes of preoperative anxiety (circle one)**

| **Code** | **Questioner** | **characteristics** |
| --- | --- | --- |
| 201 | Do you fear death during cesarean delivery? | 1. Yes 2. No |
| 202 | Do you Fear unexplained origin during cesarean delivery? | 1. Yes 2. No |
| 203 | Are worried about Financial loss if happens? | 1. Yes 2. No |
| 204 | Are worried about Family issues if a problem happens? | 1. Yes 2. No |
| 205 | Do you fear Postoperative pain? | 1. Yes 2. No |
| 206 | Are you fear of dependency if any problem happens? | 1. Yes 2. No |
| 207 | Do you fear disability due to any cause of the procedure? | 1. Yes 2. No |
| 208 | Do you fear complications with the procedure? | 1. Yes 2. No |
| 209 | Are you worried about the cosmetic issues of the scar? | 1. Yes 2. No |
| 210 | Are you worried about medical mistakes? | 1. Yes 2. No |
| 211 | Do you fear of unable to recover from anesthesia? | 1. Yes 2. No |
| 212 | Do you fear awareness during anesthesia & Cesarean delivery | 1. Yes 2. No |
| 213 | Do you Fear experience with a gynecologist/ISO? | 1. Yes 2. No |
| 214 | Do you Fear of experience an anesthetist? | 1. Yes 2. No |
| 215 | Are you worried about information from previous negative hospital experiences? | 1. Yes 2. No |
| 216 | Do you worry about the lack of recognition of staff? | 1. Yes 2. No |
| 217 | Do you worry about the bad obstetric history of previous delivery? | 1. Yes 2. No |

**III: -**Trait- State-Trait Anxiety Inventory (STAI)

Negative Items for Trait -State trait anxiety inventory (circle one)

|  | Items | Almost never | Sometimes | often | Almost always |
| --- | --- | --- | --- | --- | --- |
| 301 | I FEEL TENSE | 1 | 2 | 3 | 4 |
| 302 | I FEEL UPSET | 1 | 2 | 3 | 4 |
| 303 | I FEEL WORRIED | 1 | 2 | 3 | 4 |

**Positive Items for Trait-State trait anxiety inventory**(circle one)

|  | ITEMS | Almost never | Sometimes | often | Almost always |
| --- | --- | --- | --- | --- | --- |
| 304 | I FEEL CALM | 4 | 3 | 2 | 1 |
| 305 | I FEEL RELAXED | 4 | 3 | 2 | 1 |
| 306 | I FEEL CONTENT | 4 | 3 | 2 | 1 |

| 307 | Total STAI Score | To calculate sum up both +ve and -ve items, then multiply by 20/6. | 20 up to 80 |  |
| --- | --- | --- | --- | --- |
